# The Disease and Economic Burdens of Esophageal Cancer in China from 2013 to 2030: Dynamic Cohort Modeling Study

**DOI:** 10.2196/33191

**Published:** 2022-03-02

**Authors:** Yuanyuan Li, Junfang Xu, Yuxuan Gu, Xueshan Sun, Hengjin Dong, Changgui Chen

**Affiliations:** 1 Department of Science and Education Hangzhou Ninth People’s Hospital Hangzhou China; 2 Center for Health Policy Studies School of Public Health Zhejiang University School of Medicine Hangzhou China; 3 General Practice Hangzhou Ninth People’s Hospital Hangzhou China

**Keywords:** esophageal cancer, disease burden, disability-adjusted life year, economic burden

## Abstract

**Background:**

Esophageal cancer (EC) is the sixth leading cause of tumor-related deaths worldwide. Estimates of the EC burden are necessary and could offer evidence-based suggestions for local cancer control.

**Objective:**

The aim of this study was to predict the disease burden of EC in China through the estimation of disability-adjusted life years (DALYs) and direct medical expenditure by sex from 2013 to 2030.

**Methods:**

A dynamic cohort Markov model was developed to simulate EC prevalence, DALYs, and direct medical expenditure by sex. Input data were collected from the China Statistical Yearbooks, Statistical Report of China Children’s Development, World Population Prospects 2019, and published papers. The JoinPoint Regression Program was used to calculate the average annual percentage change (AAPC) of DALY rates, whereas the average annual growth rate (AAGR) was applied to analyze the changing direct medical expenditure trend over time.

**Results:**

From 2013 to 2030, the predicted EC prevalence is projected to increase from 61.0 to 64.5 per 100,000 people, with annual EC cases increasing by 11.5% (from 835,600 to 931,800). The DALYs will increase by 21.3% (from 30,034,000 to 36,444,000), and the years of life lost (YLL) will account for over 90% of the DALYs. The DALY rates per 100,000 people will increase from 219.2 to 252.3; however, there was a difference between sexes, with an increase from 302.9 to 384.3 in males and a decline from 131.2 to 115.9 in females. The AAPC was 0.8% (95% CI 0.8% to 0.9%), 1.4% (95% CI 1.3% to 1.5%), and –0.7% (95% CI –0.8% to –0.7%) for both sexes, males, and females, respectively. The direct medical expenditure will increase by 128.7% (from US $33.4 to US $76.4 billion), with an AAGR of 5.0%. The direct medical expenditure is 2-3 times higher in males than in females.

**Conclusions:**

EC still causes severe disease and economic burdens. YLL are responsible for the majority of DALYs, which highlights an urgent need to establish a beneficial policy to reduce the EC burden.

## Introduction

Esophageal cancer (EC) is a serious malignant tumor that originates from the esophageal epithelium. According to 2020 Global Cancer Statistics, EC is the sixth leading cause of death and the seventh most common cancer worldwide [1]. The incidence of EC has a considerable disparity in geographical distribution, with the highest EC burden in East Asia, and China had the highest number of new EC cases and deaths in 2017 [2,3]. In China, EC ranked as the fourth leading cause of death and the fifth most common cancer in 2012 [4]. There were an estimated 286,700 new cases and 210,900 deaths in 2012, with incidence and mortality rates of 21.2/10^5^ and 15.2/10^5^, respectively [5,6]. Although EC incidence and mortality have decreased, EC still causes a huge burden on society, families, and individuals, with new cases and deaths increasing by 52.3% and 40.0%, respectively, from 1990 to 2017 in China [1]. More specifically, in China, over 90% of EC patients are diagnosed with invasive cancer, 20% of them have already developed distant metastasis, and the overall 5-year survival rate is approximately 18.4% [6,7].

In China, morbidity and mortality due to EC have been well-documented; however, its prevalence, premature death rate, and disability rate, and their estimates in the next decades, have not yet been investigated in detail. Moreover, over the past decades, fewer data have been available on the estimation of direct medical expenditure caused by EC [8,9]. The existing studies mostly focus on the average direct medical expenditure per patient, the average per hospital visit, or the average daily expenditure per patient [10]. However, little is known about the total direct medical expenditure and its development trends due to EC. “Health China 2030” released the key healthy indicators that life expectancy will increase by 3 years and reach 79 years by 2030, the premature death rate caused by major chronic diseases will decline by 30%, and the proportion of personal health expenditure among the total health expenditure will decline by 25% [11]. All of these factors put forward an even greater need for the prevention and control of chronic diseases.

In this study, we estimated the EC prevalence, premature death rate, and disability rate, as well as the disability-adjusted life years (DALYs) rate and direct medical expenditure from 2013 to 2030 by sex. We provide the average annual percentage change (AAPC) of DALY rates and the average annual growth rates (AAGR) of direct medical expenditure. We hope that our study will offer policymakers evidence-based suggestions for the precise prevention and control of EC, and help to reduce the societal and economic burdens of EC in China.

## Methods

### Data Source

We chose the year 2012 as the baseline year. The birth rate, all-cause mortality, sex ratio, and standard life expectancy at birth from 2012 to 2019 were collected from the China Statistical Yearbook released by the Bureau of Statistics of China [12]. Infant mortality from 2012 to 2019 was drawn from the Statistical Report of China Children’s Development (2011-2020) [13]. All cohort data for subsequent years were drawn from the World Population Prospects 2019 released by the United Nations Population Division [14]. The birth rate and infant mortality for boys and girls were calculated for both sexes combined according to the sex ratio at birth. All-cause mortality for males and females was computed by the mortality of both sexes combined with the sex ratio of the total population and the risk ratio of all-cause mortality in males compared to females obtained from the Tabulation on the 2010 Population Census of the People’s Republic of China [15].

We used an age-specific population and all-cause mortality, age-specific EC incidence, mortality in 2012, and EC fatalities to estimate the EC prevalence in 2012, average age of onset, and duration of EC through the DisMod model [16]. Age-specific population and all-cause mortality were estimated using the data collected from the 2010 Population Census of the People’s Republic of China. EC incidence and mortality were collected from published data [6,17], whereas the fatality was estimated based on survival data [6]. Additionally, the AAPC of EC incidence and mortality were used to simulate the subsequent yearly incidence and mortality of EC [6,17].

### Measures

#### Calculation of DALYs

DALYs are a time-based measure of the overall burden of disease, representing the sum of the years of life lost (YLL) due to premature mortality and years lived with a disability (YLD) due to disease. One DALY represents a loss of the equivalent of 1 year of full health [18]. The calculations are as follows [19]:

DALY=YLL+YLD

YLL=KCe^γα^/(γ+β)^2^ {e^–(γ+β)(L+α)^ [–(γ+β)(L+α)–1] – e^–(γ+β)α^ [–(γ+β)α–1]} + 1–K/γ (1–e^–γL^)^4^

YLD=DKCe^γα′^ {e^–(γ+β)(L′+α′)^ [–(γ+β)(L′+α′)–1] – e^–(γ+β)α′^ [–(γ+β)α′–1]} + D(1–D)/γ (1–e^–γL′^)^4^

where *K* is the age-weighting modulation factor, *C* is a necessary constant to adjust for unequal age weights, *γ* is the standard discount rate, *β* is the standard age weight, *L* is the standard expectation of life at age *α*, *α* is the age at death, *D* is the disability weight, *α′* is the age of onset of the disability, and *L′* is the duration of the disability. The key parameters were set as follows: *K*=1, *C*=0.1658, *γ*=0.03, and *β*=.04. *L* is the difference between the standard life expectancy at birth and the average age at death due to EC, *α* is defined as the sum of the average age at onset and the duration of EC, and *D* was estimated using values addressed in the Disability Weights for the Global Burden of Disease (GBD) 2013 study and clinical stage data for China. According to the GBD report, the disability weights of EC diagnosis/therapy and control (stage I-II), cancer with preterminal metastasis (stage III), and the terminal phase (IV) are 0.288, 0.451, and 0.540, respectively [20]. EC clinical stage data were collected from a 10-year multicenter retrospective survey, and the proportions of stage I-II, stage III, and stage IV disease were 45.5%, 33.8%, and 20.7%, respectively [7]. Consequently, the weighted average disability weight was 0.395. Additionally, EC prevalence was used to calculate the YLD.

#### Direct Medical Expenditure Per Patient

Direct medical expenditure represents the total direct medical expenditure that occurs in the hospital. The annual average direct medical expenditure per patient was estimated using the following formula: *C*_2012_×(1+*AAGR*)*^N^*^+1^, where *C*_2012_ is the annual average direct medical expenditure per patient in 2012 that was collected from the China Health Statistics Yearbook 2013 and AAGR was estimated using the average annual direct medical expenditure per patient from 2010 to 2017 that was released by the China Health Statistics Yearbook 2010-2017 (see Table S1 in Multimedia Appendix 1) [21]. The AAGR is simply calculated by the annual percentage growth divided by the number of years using the following formula:

AAGR=∑[V_ending_/V_beginning_]–1]×100%/N–1,

where *V_ending_* represents the future value, *V_beginning_* represents the present value, and *N* represents the number of years. Additionally, the AAGR was converted to US $ using purchasing power parities of 3.506 in 2017 [22].

### Statistical Analysis

#### Cohort Markov Model

We constructed a dynamic cohort Markov model with five states, including newborn, health, EC, EC-specific death, and other deaths. We assumed EC-specific death as the nonabsorbing state. Figure S1 in Multimedia Appendix 1 displays the model, with arrows representing the transition of the cohort population between states that was determined by the predefined transition probabilities. A cycle of 1 year was chosen.

The transition from newborn to health was estimated using the following formula: T__newtohealth_=R__b_×Count_alive×(1–M__Inf_), where T__newtohealth_ represents the annual number of people transferred from the state of newborn to the health state, R__b_ is the annual birth rate, Count_alive is the number of people still alive at the end of the last simulation cycle summed by the number of people in the health state and EC state, and M__Inf_ is the annual infant death probability. The number of people transferred from the state of newborn to the state of other death is equal to 1–T__newtohealth_. We used the EC annual incidence probability to determine the number of people transferred from the health state to the EC state. The annual death probability for health was defined as the non-EC annual death probability, which was calculated by the difference between all-cause death probability and EC-related death probability. The annual death probability of the EC state was composed of two parts: the annual EC fatality probability and the non-EC death probability.

Additionally, we examined the AAPC of DALY rates, including the 95% CIs, as well as the AAGR of direct medical expenditure. TreeAge Pro 2019 was used to estimate DALYs and direct medical expenditure, and JoinPoint Regression Program software (version 4.7.0.0) was used to calculate the AAPC of DALY rates and the 95% CIs [23,24]. Other analyses were performed using Stata 14.0.

#### Sensitivity Analysis

One-way sensitivity analyses were performed to estimate the extent to which the model’s calculations were affected by the uncertainty of the input data. Sensitivity analyses were confined to the birth rate and all-cause mortality for both sexes, the sex ratio of the total population, the AAPC for EC incidence and mortality, the 5-year survival rate, and the AAGR of the direct medical expenditure. The ranges of the birth rate, all-cause mortality, and sex ratio of the total population were derived from World Population Prospects 2019. The ranges of the AAPC for EC incidence and mortality and the 5-year survival rate were the 95% CIs collected from published papers. The range of the AAGR for direct medical expenditure was assumed to be simply a “plausible” range, which varied by ±10% of the base case value.

#### Validation

We performed the internal validation by comparing the input EC incidence with the simulated EC incidence by the model. The goodness of fit was computed by plotting the model predictions versus the inputs and fitting a linear curve without an intercept. The squared linear correlation coefficient (*R^2^*) simulated by linear regression was used to examine the goodness of fit of the model.

## Results

### Internal Validation

The simulated EC incidence by the model closely matched the input data (Figure S2 in Multimedia Appendix 1). The regression line slope was 1.01 and the *R^2^* was 0.99, which demonstrated good consistency between the two estimates.

### Predicted EC Prevalence and the Number of People With EC

From 2013 to 2030, the prevalence of EC will increase from 61.00 to 64.5 per 100,000 and peak at 67.9 per 100,000 in 2020. In males, there was a dramatic increase predicted for EC incidence, from 82.0 to 96.4 per 100,000, whereas the incidence is predicted to first increase and then decrease in females, resulting in an overall decrease from 38.9 to 31.6 per 100,000. The total EC cases will increase by 11.5% (from 835,600 to 931,800), and will peak at 961,500 in 2021. The EC cases in males will increase by 22.9% (from 575,500 to 707,500), whereas in females, it will first increase and then decrease, with an overall decrease of 13.8% (from 260,100 to 224,3000). Table 1 summarizes the details.

**Table 1:** Predicted prevalence of esophageal cancer and the number of people diagnosed with esophageal cancer from 2013 to 2030 in China, by sex.

Year	Males			Females			Total	
	Prevalence (per 100,000)	Cases, n	Prevalence (per 100,000)		Cases, n	Prevalence (per 100,000)		Cases, n
2013	82.0	575,500	38.9		260,100	61.0		835,600
2014	86.1	607,600	40.4		270,900	63.8		878,500
2015	89.0	631,100	41.1		277,000	65.6		908,100
2016	91.1	648,600	41.3		279,900	66.8		928,500
2017	92.4	661,900	41.1		280,500	67.4		942,300
2018	93.4	672,300	40.7		279,400	67.7		951,600
2019	94.3	680,600	40.2		277,100	67.9		957,700
2020	94.9	687,100	39.6		273,800	67.9		961,000
2021	95.3	691,800	38.9		269,800	67.7		961,500
2022	95.6	695,500	38.1		265,300	67.5		960,700
2023	95.8	698,500	37.3		260,500	67.2		959,000
2024	95.9	701,100	36.5		255,600	66.8		956,600
2025	96.0	703,400	35.6		250,500	66.4		953,900
2026	96.1	704,700	34.8		245,300	66.0		950,000
2027	96.2	705,700	34.0		240,000	65.7		945,700
2028	96.3	706,500	33.2		234,700	65.3		941,200
2029	96.4	707,100	32.4		229,500	64.9		936,500
2030	96.4	707,500	31.6		224,300	64.5		931,800

### DALYs, the DALY Rate, and the AAPC

**Figure 1:**
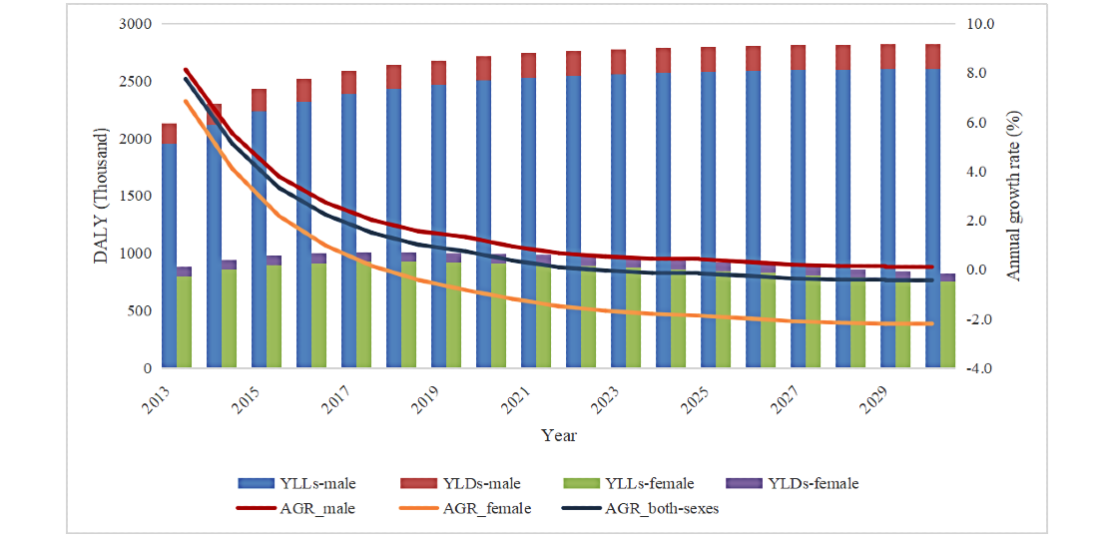
The predicted disability-adjusted life years (DALYs) of esophageal cancer by sex from 2013 to 2030. YLL: years of life lost; YLD: years lived with a disability; AGR: annual growth rate.

**Figure 2:**
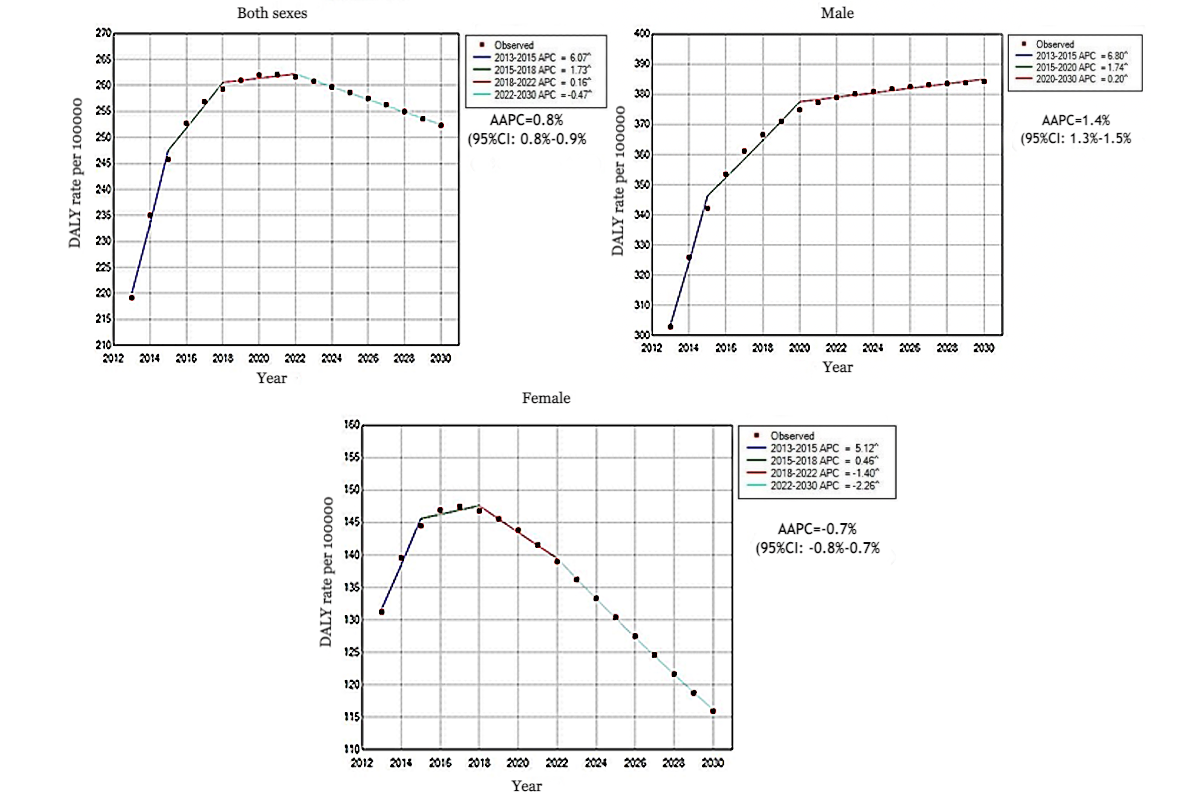
The predicted changing trend of disability-adjusted life years (DALYs) due to esophageal cancer from 2013 to 2030 in China, by sex. In all 3 graphs, the Annual Percent Change (APC) and the Average Annual Percentage Change (AAPC) are significantly different from zero at *α*=.05 level.

Figure 1 presents the DALYs due to EC by sex in China between 2013 and 2030. From 2013 to 2030, the total DALYs for both sexes will increase by 21.3% (from 30,034,000 to 36,444,000). DALYs will increase by 32.6% in males (from 21,266,000 to 28,209,000); in contrast, they will decrease overall by 6.1% in females (from 876,800 to 823,500). Significantly, YLL contributed to over 90% of the DALYs for both sexes. Figure 2 displays the DALY rates per 100,000 people by year and sex.

The results of the Joinpoint regression analysis address the statistical significance of the AAPC in DALY rates. From 2013 to 2030, the overall DALY rates per 100,000 will increase from 219.2 to 252.3, and will peak at 262.1 in 2021. The AAPC will be 0.8% (95% CI 0.8%-0.9%). In males, there was an obvious increase from 302.9 to 384.3, with 1.4% (95% CI 1.3%-1.5%) of the AAPC. However, an overall significant downward trend was shown in females (from 131.2 to 115.9), which peaked at 147.4 in 2017. The AAPC will be –0.7% (95% CI –0.8% to –0.7%).

### Projected Annual Direct Medical Expenditure

**Figure 3:**
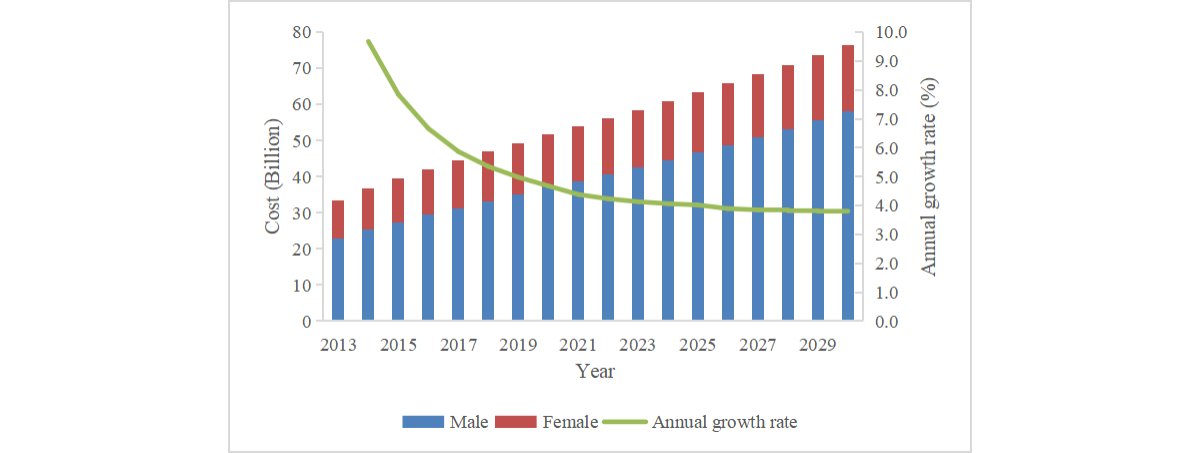
Estimated total annual direct medical expenditure of esophageal cancer from 2013 to 2030 in China, by sex.

The predicted annual direct medical expenditure for both sexes will increase significantly (Figure 3). From 2013 to 2030, the total annual direct medical expenditure will increase by 128.7% (from US $33.4 billion to US $76.4 billion). The AAGR will be 5.0%, and the annual growth rate will decline from 9.7% to 3.8%. Male patients will have 2.2-3.2 times higher direct medical expenditure than female patients, which will increase by 152.2% and 76.9% (from US $23.0 billion to US $58.0 billion and from US $10.4 billion to US $18.4 billion) in males and females, respectively.

### Sensitivity Analysis

The results of the sensitivity analysis indicated that changes in the AAGR of direct medical expenditure, 5-year survival rate, and AAPC of EC incidence have a substantial impact on the direct medical expenditure of EC, whereas the disability weight, 5-year survival rate, AAPC of EC incidence, risk ratio of all-cause mortality, and birth rate have an obvious impact on the DALYs. Table 2 and Table 3 summarize the changes in the cumulative direct medical expenditure and DALYs due to EC according to the variations in key input data considered in the sensitivity analyses for males and females, respectively.

**Table 2:** Changes in the estimated cumulative direct medical expenditure (DME) and disability-adjusted life years (DALYs) of esophageal cancer in males in China according to the key parameters considered in the sensitivity analyses.

Parameter		Changes in estimated DME, billions (%)				Changes in estimated DALYs, thousands (%)		
		2030	2025	2020	2030		2025	2020
Baseline value		741.7	475.2	261.9	49957.5		35889.7	22037.9
**AAGR^a^ of DME**								
	0.0475 instead of 0.0432	33.1 (4.5)	14.8 (3.1)	4.9 (1.7)	NC^b^		NC	NC
	0.0388 instead of 0.0432	–32.2 (–4.3)	–14.7 (–3.1)	–4.9 (–1.9)	NC		NC	NC
**5-year survival rate**								
	0.208 instead of 0.199	12.3 (1.7)	7.2 (1.5)	3.3 (1.3)	–130.8 (–0.3)		–139.3 (–0.4)	–135.9 (–0.6)
	0.190 instead of 0.199	–12.4 (–1.7)	-7.3 (-1.5)	–3.4 (–1.3)	131.0 (0.3)		139.9 (0.4)	137.5 (0.6)
**Risk ratio of all-cause mortality**								
	1.47 instead of 1.33	–2.7 (–0.4)	–1.2 (–0.3)	–0.4 (–0.2)	–146.0 (–0.3)		–71.5 (–0.2)	–24.4 (–0.1)
	1.20 instead of 1.33	2.8 (0.4)	1.2 (0.3)	0.4 (0.2)	153.2 (0.3)		74.9 (0.2)	25.5 (0.1)
**Birth rate**								
	High estimates^c^	2.6 (0.4)	0.3 (0.1)	NC	107.2 (0.2)		12.14 (0.0)	NC
	Low estimates^c^	–2.6 (–0.4)	–0.3 (–0.1)	NC	–107.6 (–0.2)		-12.2 (0.0)	NC
**Sex ratio of the total population**								
	High estimates^d^	–0.1 (0.0)	–0.01 (0.0)	NC	–5 (0.0)		–0.5 (0.0)	NC
	Low estimates^d^	0.1 (0.0)	NC	NC	5.0 (0.0)		0.6 (0.0)	NC
**Risk ratio of infant mortality**								
	1.04 instead of 0.95	NC	NC	NC	–1.2 (0.0)		0.7 (0.0)	–0.2 (0.0)
	0.85 instead of 0.94	NC	NC	NC	1.5 (0.0)		–0.6 (0.0)	0.2 (0.0)
**Disability weight**								
	0.435 instead of 0.395	NC	NC	NC	393.2 (0.8)		284.0 (0.8)	176.1 (0.8)
	0.356 instead of 0.395	NC	NC	NC	–393.2 (–0.8)		–284.0 (–0.8)	–176.1 (–0.8)
**All-cause mortality**								
	High estimates^e^	NC	NC	NC	NC		NC	NC
	Low estimates^e^	NC	NC	NC	NC		NC	NC

^a^AAGR: average annual growth rate.

^b^NC: no changes in estimated costs or DALYs.

^c^See Table S2 in Multimedia Appendix 1.

^d^See Table S3 in Multimedia Appendix 1.

^e^See Table S4 in Multimedia Appendix 1.

**Table 3:** Changes in the estimated cumulative costs and disability-adjusted life years (DALYs) of esophageal cancer (EC) in females in China according to the key parameters considered in the sensitivity analyses.

Parameter			Changes in estimated cost, billions(%)						Changes in estimated DALYs, thousands (%)			
			2030		2025		2020	2030			2025	2020
Baseline value			279.5		191.1		111.7	17687.7			13382.4	8628.1
**AAGR^a^ of DME^b^**												
	0.0475 instead of 0.0432	11.8 (4.2)		5.8 (3.0)		2.0 (1.8)		NC^c^		NC		NC
	0.0388 instead of 0.0432	–11.4 (–4.1)		–5.7 (–3.0)		–2.0 (–1.8)		NC		NC		NC
**AAPC^d^ of EC incidence**												
	–0.016 instead of –0.025	18.3 (6.5)		7.7 (4.0)		2.1 (1.9)		873.3 (5.0)		402.4 (3.0)		109.1 (1.3)
	–0.035 instead of –0.025	–18.4 (–6.6)		–8.0 (–4.2)		–2.2 (–2.0)		–891.9 (–5.0)		–422.9 (–3.2)		–117.9 (–1.4)
**AAPC of EC mortality**												
	–0.011 instead of –0.027	NC		NC		NC		0.9 (0.0)		0.4 (0.0)		0.1 (0.0)
	–0.042 instead of –0.027	NC		NC		NC		–0.8 (0.0)		–0.3 (0.0)		–0.1 (0.0)
**5-year survival rate**												
	0.208 instead of 0.199	6.9 (2.5)		4.2 (2.2)		2.0 (1.8)		–57.9 (–0.3)		–71.8 (–0.5)		–77.5 (–0.9)
	0.190 instead of 0.199	–6.4 (–2.3)		–4.0 (–2.1)		–1.9 (–1.7)		53.3 (0.3)		66.6 (0.5)		72.9 (0.8)
**Risk ratio of all-cause mortality**												
	1.47 instead of 1.33	1.0 (0.4)		0.8 (0.2)		0.2 (0.2)		51.2 (0.3)		27.0 (0.2)		10.0 (0.1)
	1.20 instead of 1.33	–1.1 (–0.4)		–0.5 (–0.3)		–0.2 (–0.2)		–53.3 (–0.3)		–28.1 (–0.2)		–10.3 (–0.1)
**Birth rate**												
	High estimates^e^	0.7 (0.3)		0.1 (0.1)		NC		27.0 (0.2)		3.2 (0.0)		NC
	Low estimates^e^	–0.7 (–0.3)		–0.1 (–0.1)		NC		–27.2 (–0.2)		–3.2 (0.0)		NC
**Sex ratio of the total population**												
	High estimates^f^	NC		NC		NC		–1.2 (–0.0)		–0.1 (0.0)		NC
	Low estimates^f^	NC		NC		NC		1.2 (0.0)		0.1 (0.0)		NC
**Risk ratio of infant mortality**												
	1.04 instead of 0.95	NC		NC		NC		0.4 (0.0)		0.19 (0.0)		0.1 (0.0)
	0.85 instead of 0.94	NC		NC		NC		–0.5 (0.0)		–0.2 (0.0)		–0.1 (0.0)
**Disability weight**												
	0.435 instead of 0.395	NC		NC		NC		147.6 (0.8)		112.3 (0.9)		73.3 (0.9)
	0.356 instead of 0.395	NC		NC		NC		–147.6 (–0.8)		–112.3 (–0.8)		–73.3 (–0.9)
**All-cause mortality**												
	High estimates^e^	NC		NC		NC		NC		NC		NC
	Low estimates^e^	NC		NC		NC		NC		NC		NC

^a^AAGR: average annual growth rate.

^b^DME: direct medical expenditure.

^c^NC: no changes in estimated costs or DALYs.

^d^AAPC: average annual percentage change.

^e^See Table S2 in Multimedia Appendix 1.

^f^See Table S3 in Multimedia Appendix 1.

## Discussion

### Principal Findings

To the best of our knowledge, this is the first modeling study on the national disease burden of EC in China. According to our estimates, the disease and economic burdens due to EC will increase substantially over the next few decades. From 2013 to 2030, the EC prevalence will increase by 11.5% (from 835,600 to 931,800); the total DALYs will increase by 21.3% (from 30,034,000 to 36,444,000); the DALY rates per 100,000 people will increase from 219.2 to 252.3, with an AAPC of 0.8%; and the direct medical expenditure will increase by 128.7% (from US $33.4 billion to US $76.4 billion), with an AAGR of 5%. Additionally, we found large variations in burdens between sexes: the prevalence and direct medical expenditure was 2.2-3.2 times higher and DALYs were 2.4-3.4 times higher in males than in females.

China ranks close to the top in terms of the highest number of EC incident cases, deaths, and DALYs worldwide [2]. The DALY rates per 100,000 in 2017 were twice as high as those in the rest of the world (222.6 vs 119.9) [2]. This is largely because of the higher aging population, low social demographic index, and high-risk lifestyles related to EC in China. Our estimated DALY rates in 2017 were slightly higher than the estimates provided by the GBD 2017 Esophageal Cancer Collaborators (256.9 vs 222.6). This discrepancy is probably due to the different sources of the data used for calculating the DALYs. Almost all of the DALYs were attributed to YLL due to premature death, which is quite consistent with previous studies [19,25,26]. Additionally, the burden from EC in China has geographic disparities, and high-risk areas cause an almost 4-times higher burden than that estimated at the national level (810.0 vs 219.2 per 100,000) [19].

Despite previous studies indicating a decrease in the incidence and mortality of EC over the past decades, this study suggests a continuous increase in the prevalence of EC, DALYs, and direct medical expenditure. This is driven primarily by the increasing absolute number of EC cases. Additionally, the sensitivity analysis demonstrated that the incidence of EC, 5-year survival rate, and disability weight have a considerable impact on DALYs. Collectively, these findings suggest the significance of reducing the incidence of EC, premature death, and personal direct medical expenditure, and improving the 5-year survival rate and disease health-related quality of life.

Consequently, policymakers should adopt comprehensive effective strategies to reduce the burden caused by EC, possibly focusing on the consciousness of cancer prevention, early detection, early diagnosis, and early treatment, as well as the demand for and access to cancer prevention and treatment knowledge. First, screening should be highly recommended since it is the major strategy for primary and secondary disease prevention, and could detect the disease in the early stage to treat it effectively [27]. Additionally, considering the cost-effectiveness and the large disparities according to age, sex, and other higher-risk behaviors, a finer delineation of strategies should be specified for individuals at different risk levels [28,29]. Second, improving health literacy, including personal health literacy and organizational health literacy, is positively related to awareness of cancer risk and knowledge, health behavior and screening, early cancer symptom recognition, cancer screening behavior, health service resource utilization, treatment compliance, and quality of life [30]. Moreover, educational interventions using social media should be suggested to promote self-management and strengthen health literacy [31,32]. Furthermore, clinical treatment and terminal care should be strengthened to improve the quality of life and 5-year survival rate.

China has suffered a high catastrophic health expenditure (CHE). The total out-of-pocket payment expenses accounted for approximately 30% of the total health expenditure, increasing by 120.6% between 2010 and 2019 [33]. The average incidence of CHE in recent decades was 23.3% (95% CI 21.1%-25.6%) [34], whereas it was 60.1% in cancer patients [27,35]. The overall incidence of CHE in EC patients was 69.2%, followed by colon cancer, breast cancer, and liver cancer [27]. Specifically, CHE is closely linked with health insurance, which reached 73.6% and 100% in patients under the new rural cooperative medical scheme and in patients without coverage by health insurance, respectively [27]. Although the predicted annual growth rate of direct medical expenditure due to EC will decline and the proportion of direct medical expenditure among total health expenditure will decrease from 0.47% to 0.36% between 2013 and 2017, the estimated absolute direct medical expenditure will still increase over the next decades [33]. Thus, EC remains a severe economic burden. Moreover, esophageal squamous cell carcinoma (ESCC) is the dominant histological subtype of EC in China, and studies have shown a strong inverse relationship between ESCC and socioeconomic status [2,36,37]. Financial capability largely determines the treatment and health service utilization for cancer patients in China. The current insurance schemes are insufficient to address these disparities [38]. All of these findings imply that more integrated strategies are needed, including improving a household’s economic level, expanding the breadth of insurance coverage, reinforcing prepayment hospital insurance methods, and strengthening the public assistance system. Additionally, the scope of national drug centralized volume–based procurement should be expanded, since it has substantial importance in reducing medical costs and the expenditure of medical insurance funds, saving procurement funds, and reducing the patients’ burdens due to use of the drug [39,40].

### Limitations

Some limitations should be noted when interpreting our results. First, it is difficult to examine the external validation of our model since there are no comparable published data. However, our model has good face and internal validity because it was developed based on the structure of the DisMod II model, which presents the dynamic development of EC very well, and goodness-of-fit analyses showed the internal validity. Second, our model underestimates the total economic burden of EC, since the indirect and hidden costs were not simulated. Third, the input data were estimated based on past data values, which cannot provide accurate evaluations. However, the sensitivity analysis presented the changes caused by the uncertainty of the inputs. Moreover, the goodness-of-fit analysis showed the good internal validity of the model.

### Conclusion

EC still causes serious disease and economic burdens in China, and DALYs and direct medical expenditures will continue to increase over the next decades. Male patients have a higher burden than female patients. YLL account for the majority of DALYs. Comprehensive strategies should be implemented for the improvement of EC prevention and control, including screening, improving health literacy, improving a household’s economic level, expanding the breadth of insurance coverage, reinforcing prepayment hospital insurance methods, strengthening the public assistance system, and expanding the scope of national drug centralized volume–based procurement. In addition, clinical treatment and terminal care should be strengthened to improve quality of life.

## Appendix

Multimedia Appendix 1 Supplementary data (Figures S1-S2, Tables S1-S11).

## Abbreviations

AAGR: average annual growth rate
AAPC: average annual percentage change
CHE: catastrophic health expenditure
DALY: disability-adjusted life year
EC: esophageal cancer
ESCC: esophageal squamous cell carcinoma
GBD: Global Burden of Disease
YLD: years of life lost due to disability
YLL: years of life lost due to premature mortality
