# Supplementary material for: The Disease and Economic Burdens of Esophageal Cancer in China from 2013 to 2030: Dynamic Cohort Modeling Study
Source: JMIR Public Health Surveill. 2022 Mar 2;8(3):e33191. doi: 10.2196/33191 (PMC8928052; doi:10.2196/33191)
Supplement: Multimedia Appendix 1 [file publichealth_v8i3e33191_app1.docx]

**Supplementary data**

Figure S1 Dynamic Cohort esophageal cancer Markov Model with five states

Figure S2 Model internal validation by goodness-of-fit test

Table S1 The average annual direct medical expenditure per patient from 2010 to 2017

Table S2 The birth rate for both sexes by years

Table S3 Sex ratio of the total population and at birth by years

Table S4 All-cause mortality and infant mortality for both sexes by years

Table S5 Standard Life expectancy at birth for both sexes, males and females by years

Table S6 Other parameters in the model

Table S7 Age specific mortality in China in 2012

Table S8 Age specific population in China in 2010

Table S9 Age specific incidence rate of esophagus cancer in China in 2012

Table S10 Age specific mortality of esophagus cancer in China in 2012

Table S11 The predicted incidence per 100 000 of esophageal cancer from model simulation and model input

**Figure S1**. Dynamic Cohort Esophageal cancer Markov Model with five states

Health

Disease-specific death

Esophageal cancer

Other death

Newborn

**Figure S2.** Model internal validation by goodness-of-fit test

**
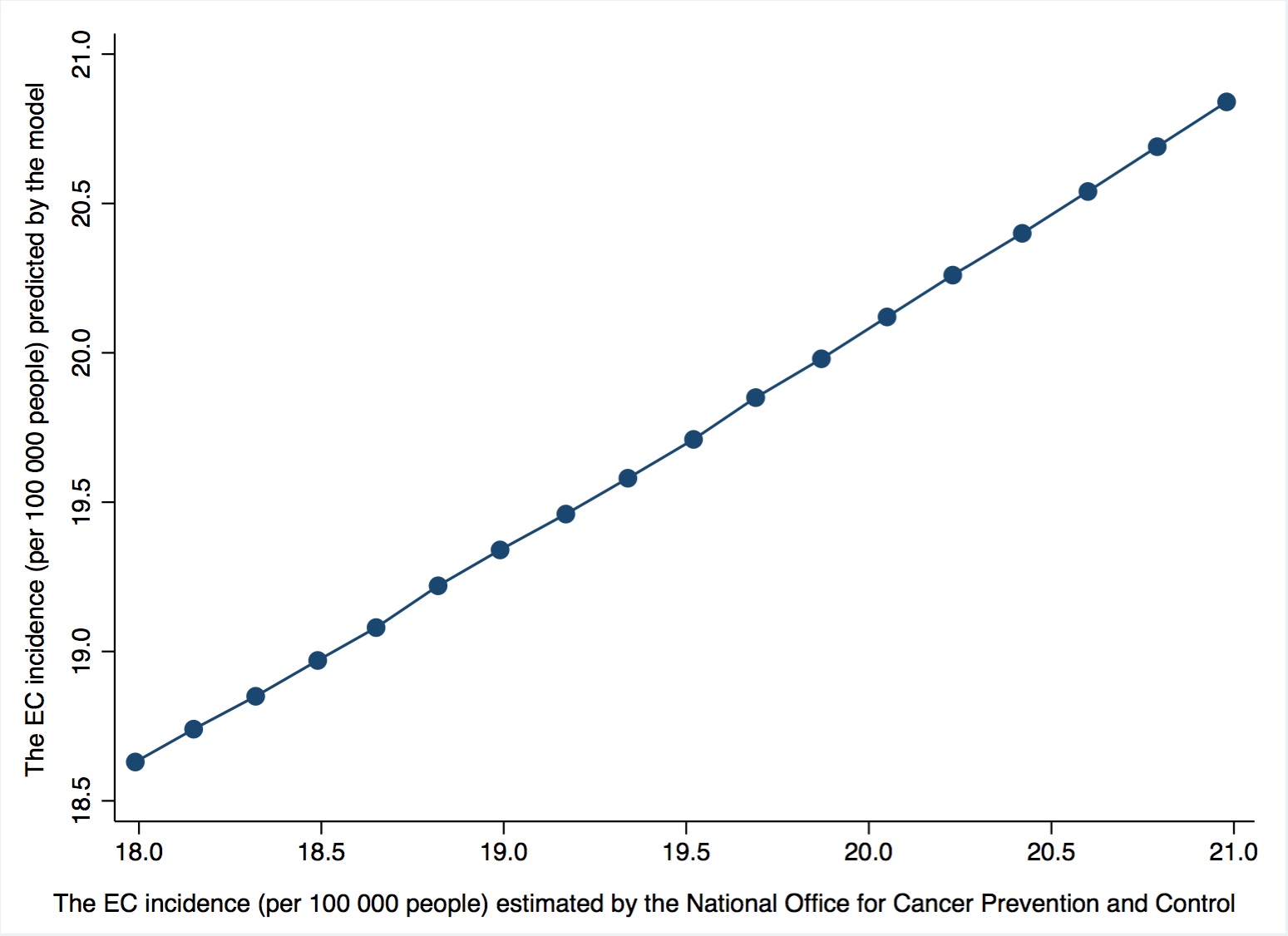
**

**Table S1.** The average annual direct medical expenditure per patient from 2010 to 2017 (USD)

| Year | Inpatient costs | Outpatient fee | Total |
| --- | --- | --- | --- |
| 2017 | 5473.2 | 73.3 | 5546.5 |
| 2016 | 5121.4 | 70.3 | 5191.8 |
| 2015 | 5044.7 | 67.1 | 5111.8 |
| 2014 | 4899.3 | 63.2 | 4962.5 |
| 2013 | 4808.7 | 59.3 | 4868.0 |
| 2012 | 3774.1 | 55.2 | 3829.2 |
| 2011 | 4173.2 | 51.4 | 4224.6 |
| 2010 | 4075.1 | 49.6 | 4124.7 |

Note: The average annual direct medical expenditure per patient was changed into US dollars using the purchasing power parities with 3.506 in 2017.

**Table S2.** The birth rate for both sexes by years

| Year | Low* | Estimates | High* | Reference |
| --- | --- | --- | --- | --- |
| 2013 | 0.012080 | 0.012080 | 0.012080 | [12] |
| 2014 | 0.012370 | 0.012370 | 0.012370 |  |
| 2015 | 0.012070 | 0.012070 | 0.012070 |  |
| 2016 | 0.012950 | 0.012950 | 0.012950 |  |
| 2017 | 0.012430 | 0.012430 | 0.012430 |  |
| 2018 | 0.010940 | 0.010940 | 0.010940 |  |
| 2019 | 0.010480 | 0.010480 | 0.010480 |  |
| 2020 | 0.009119 | 0.010645 | 0.012159 | [14] |
| 2021 | 0.009119 | 0.010645 | 0.012159 |  |
| 2022 | 0.009119 | 0.010645 | 0.012159 |  |
| 2023 | 0.009119 | 0.010645 | 0.012159 |  |
| 2024 | 0.009119 | 0.010645 | 0.012159 |  |
| 2025 | 0.007624 | 0.009808 | 0.011934 |  |
| 2026 | 0.007624 | 0.009808 | 0.011934 |  |
| 2027 | 0.007624 | 0.009808 | 0.011934 |  |
| 2028 | 0.007624 | 0.009808 | 0.011934 |  |
| 2029 | 0.007624 | 0.009808 | 0.011934 |  |
| 2030 | 0.006900 | 0.009460 | 0.011891 |  |

Note: The birth rate from 2012 to 2019 was collected from China Statistical Yearbook; The birth rate and their low and high value between 2020 and 2030 was collected from World Population Prospects 2019; * mean the values used for sensitivity analyses.

**Table S3.** Sex ratio of the total population and at birth by years

| Year | Sex ratio of the total population  (males per 100 females) | | | Sex ratio at birth  (male births per 100 female births) | Reference |
| --- | --- | --- | --- | --- | --- |
|  | Low* | Estimates | High* |  |  |
| 2013 | 105.22 | 105.22 | 105.22 | 117.70 | [12] |
| 2014 | 105.04 | 105.04 | 105.04 | 117.60 |  |
| 2015 | 105.02 | 105.02 | 105.02 | 115.88 |  |
| 2016 | 104.98 | 104.98 | 104.98 | 113.51 |  |
| 2017 | 104.81 | 104.81 | 104.81 | 112.88 |  |
| 2018 | 104.64 | 104.64 | 104.64 | 111.90 |  |
| 2019 | 104.46 | 104.46 | 104.46 | 112.00 |  |
| 2020 | 105.30 | 105.30 | 105.30 | 110.14 | [14] |
| 2021 | 105.30 | 105.30 | 105.30 | 111.00 |  |
| 2022 | 105.30 | 105.30 | 105.30 | 111.00 |  |
| 2023 | 105.30 | 105.30 | 105.30 | 111.00 |  |
| 2024 | 105.30 | 105.30 | 105.30 | 111.00 |  |
| 2025 | 104.88 | 104.92 | 104.96 | 111.00 |  |
| 2026 | 104.88 | 104.92 | 104.96 | 109.00 |  |
| 2027 | 104.88 | 104.92 | 104.96 | 109.00 |  |
| 2028 | 104.88 | 104.92 | 104.96 | 109.00 |  |
| 2029 | 104.88 | 104.92 | 104.96 | 109.00 |  |
| 2030 | 104.37 | 104.47 | 104.56 | 109.00 |  |

Note: The sex ratio from 2012 to 2019 was collected from China Statistical Yearbook, the sex ratio and their low and high value between 2020 and 2030 was collected from World Population Prospects 2019; * mean the values used for sensitivity analyses.

**Table S4.** All-cause mortality and infant mortality for both sexes by years

| Year | All-cause mortality | | | Infant mortality | Reference |
| --- | --- | --- | --- | --- | --- |
|  | Low* | Estimates | High* |  |  |
| 2013 | 0.007160 | 0.007160 | 0.007160 | 0.009500 | [12-13] |
| 2014 | 0.007160 | 0.007160 | 0.007160 | 0.008900 |  |
| 2015 | 0.007110 | 0.007110 | 0.007110 | 0.008100 |  |
| 2016 | 0.007090 | 0.007090 | 0.007090 | 0.007500 |  |
| 2017 | 0.007110 | 0.007110 | 0.007110 | 0.006800 |  |
| 2018 | 0.007130 | 0.007130 | 0.007130 | 0.006100 |  |
| 2019 | 0.007140 | 0.007140 | 0.007140 | 0.005600 |  |
| 2020 | 0.007806 | 0.007823 | 0.007839 | 0.008397 | [14] |
| 2021 | 0.007806 | 0.007823 | 0.007839 | 0.008397 |  |
| 2022 | 0.007806 | 0.007823 | 0.007839 | 0.008397 |  |
| 2023 | 0.007806 | 0.007823 | 0.007839 | 0.008397 |  |
| 2024 | 0.007806 | 0.007823 | 0.007839 | 0.008397 |  |
| 2025 | 0.008592 | 0.008686 | 0.008784 | 0.007239 |  |
| 2026 | 0.008592 | 0.008686 | 0.008784 | 0.007239 |  |
| 2027 | 0.008592 | 0.008686 | 0.008784 | 0.007239 |  |
| 2028 | 0.008592 | 0.008686 | 0.008784 | 0.007239 |  |
| 2029 | 0.008592 | 0.008686 | 0.008784 | 0.007239 |  |
| 2030 | 0.009471 | 0.009692 | 0.009925 | 0.006319 |  |

Note: The all-cause mortality from 2012 to 2019 was collected from China Statistical Yearbook, the all-cause mortality and their low and high value between 2020 and 2030 was collected from World Population Prospects 2019; The infant mortality from 2012 to 2019 was collected from statistical report of China Children’s development (2011-2020), the infant mortality between 2020 and 2030 was the calculations estimated by United Nations population division; * mean the values used for sensitivity analyses.

**Table S5.** Standard Life expectancy at birth for both sexes, males and females by years

| Year | Both sex | Males | Females | Reference |
| --- | --- | --- | --- | --- |
| 2013 | 74.83 | 72.38 | 77.37 | [12] |
| 2014 | 74.83 | 72.38 | 77.37 |  |
| 2015 | 76.34 | 73.64 | 79.43 |  |
| 2016 | 76.34 | 73.64 | 79.43 |  |
| 2017 | 76.34 | 73.64 | 79.43 |  |
| 2018 | 76.34 | 73.64 | 79.43 |  |
| 2019 | 76.34 | 73.64 | 79.43 |  |
| 2020 | 77.47 | 75.36 | 79.73 | [14] |
| 2021 | 77.47 | 75.36 | 79.73 |  |
| 2022 | 77.47 | 75.36 | 79.73 |  |
| 2023 | 77.47 | 75.36 | 79.73 |  |
| 2024 | 77.47 | 75.36 | 79.73 |  |
| 2025 | 78.31 | 76.26 | 80.47 |  |
| 2026 | 78.31 | 76.26 | 80.47 |  |
| 2027 | 78.31 | 76.26 | 80.47 |  |
| 2028 | 78.31 | 76.26 | 80.47 |  |
| 2029 | 78.31 | 76.26 | 80.47 |  |
| 2030 | 79.13 | 77.17 | 81.17 |  |

Note: The life expectancy at birth from 2012 to 2019 was collected from China Statistical Yearbook, the life expectancy at birth between 2020 and 2030 was collected from World Population Prospects 2019.

**Table S6.** Other parameters in the model

| Parameters | | | | | Resources | | |
| --- | --- | --- | --- | --- | --- | --- | --- |
| **Cohort in baseline year (2012)** | | | | | | | |
|  | | Both sex | Male | Female | |  | |
| New people | |  | 0 | 0 | | - | |
| Health | | 1353266013 | 692666146 | 660599867 | | [15] | |
| esophageal cancer | | 773987 | 531157 | 242830 | | * | |
| Disease-specific death | | 210900 | 149000 | 61900 | | [6] | |
| Other death | | 9484026 | 5462368 | 4021658 | | [15] | |
| **EC epidemiological data** | | | | | | | |
| EC incidence in 2012 | 0.000212 | | 0.000293 | 0.000127 | | | [5] |
| AAPC for EC incidence | -0.009  (-0.016- -0.001)^#^ | | 0.000 | -0.025  (-0.035--0.016)^#^ | | | [5] |
| EC Mortality in 2012 | 0.000156 | | 0.0002148 | 0.0000937 | | | [6] |
| AAPC for EC mortality | -0.011  (-0.018- -0.005)^#^ | | 0.000 | -0.027(-0.042- -0.011)^#^ | | | [6] |
| 5-year survival rate | 0.209  (0.202-0.217)^#^ | | 0.199  (0.190-0.208)^#^ | 0.236  (0.223-0.250)^#^ | | | [6] |
| Average ages at death | 68.70 | | 68.10 | 69.30 | | | * |
| Age at onset | 65.40 | | 64.80 | 66.00 | | | * |
| EC duration | 3.30 | | 3.30 | 3.30 | | | * |
| Disability weight | 0.395 | | 0.395 | 0.395 | | | [7,20] |
| **Average annual direct medical expenditure (DME) per patient** ^a^ | | | | | | | |
| Average DME in 2012 ($) | 3829.2 (3063.4-4595.1) ^b^ | | | | | [21] | |
| Average annual increase rate (AAIR) | 0.0432 (0.0388- 0.0475) ^b^ | | | | | [21] | |

Note: * predicted by the DisMod model; ^#^ mean 95% confidence interval that used for sensitivity analysis; a : the average annual direct medical expenditure per patient in 2012 was collected from the China Health Statistics Yearbook 2013, the average annual increase rate was estimated using the average annual direct medical expenditure per patient from 2010 to 2017 that was released by the China Health Statistics Yearbook 2010 – 2017 (see eTable 10) with the follow formula: AAIR= (C_2017_/C_2010_)^(1/7) -1, where C_2017_ was the average annual direct medical expenditure per patient in 2017, C_2010_ was the average annual direct medical expenditure per patient in 2010; b means the range of the costs that used for sensitivity analysis, it was assumed to be simply a “plausible” range that varied by $\pm$20%.

**Table S7.** Age specific mortality in China in 2010 (1/100 000)

| Age group | Both sexes | Male | Female |
| --- | --- | --- | --- |
| 0 | 0.0038158 | 0.0037300 | 0.0039182 |
| 1 | 0.0011126 | 0.0011598 | 0.0010557 |
| 2 | 0.0006273 | 0.0006733 | 0.0005727 |
| 3 | 0.0004516 | 0.0005011 | 0.0003930 |
| 4 | 0.0003698 | 0.0004156 | 0.0003156 |
| 5 | 0.0003332 | 0.0003706 | 0.0002889 |
| 6 | 0.0003184 | 0.0003713 | 0.0002555 |
| 7 | 0.0002849 | 0.0003465 | 0.0002118 |
| 8 | 0.0002831 | 0.0003428 | 0.0002121 |
| 9 | 0.0002819 | 0.0003492 | 0.0002023 |
| 10 | 0.0003047 | 0.0003686 | 0.0002295 |
| 11 | 0.0002926 | 0.0003509 | 0.0002243 |
| 12 | 0.0003009 | 0.0003713 | 0.0002193 |
| 13 | 0.0002940 | 0.0003621 | 0.0002159 |
| 14 | 0.0003038 | 0.0003871 | 0.0002096 |
| 15 | 0.0003441 | 0.0004468 | 0.0002308 |
| 16 | 0.0003502 | 0.0004629 | 0.0002288 |
| 17 | 0.0003901 | 0.0005198 | 0.0002507 |
| 18 | 0.0004128 | 0.0005541 | 0.0002616 |
| 19 | 0.0004263 | 0.0005940 | 0.0002519 |
| 20 | 0.0004661 | 0.0006509 | 0.0002781 |
| 21 | 0.0004722 | 0.0006585 | 0.0002845 |
| 22 | 0.0004969 | 0.0006892 | 0.0003032 |
| 23 | 0.0005370 | 0.0007495 | 0.0003252 |
| 24 | 0.0005629 | 0.0007885 | 0.0003381 |
| 25 | 0.0005829 | 0.0008163 | 0.0003485 |
| 26 | 0.0005726 | 0.0007982 | 0.0003452 |
| 27 | 0.0005940 | 0.0008137 | 0.0003700 |
| 28 | 0.0006112 | 0.0008559 | 0.0003604 |
| 29 | 0.0006812 | 0.0009430 | 0.0004131 |
| 30 | 0.0006988 | 0.0009479 | 0.0004392 |
| 31 | 0.0007697 | 0.0010539 | 0.0004754 |
| 32 | 0.0008062 | 0.0011141 | 0.0004856 |
| 33 | 0.0008284 | 0.0011460 | 0.0004960 |
| 34 | 0.0009436 | 0.0012751 | 0.0005981 |
| 35 | 0.0010271 | 0.0014193 | 0.0006187 |
| 36 | 0.0010639 | 0.0014526 | 0.0006576 |
| 37 | 0.0011351 | 0.0015493 | 0.0006997 |
| 38 | 0.0012055 | 0.0016385 | 0.0007489 |
| 39 | 0.0013412 | 0.0018379 | 0.0008208 |
| 40 | 0.0015099 | 0.0020357 | 0.0009617 |
| 41 | 0.0015530 | 0.0021014 | 0.0009821 |
| 42 | 0.0018213 | 0.0024673 | 0.0011524 |
| 43 | 0.0018879 | 0.0025685 | 0.0011828 |
| 44 | 0.0020651 | 0.0027733 | 0.0013240 |
| 45 | 0.0023140 | 0.0031042 | 0.0014915 |
| 46 | 0.0023597 | 0.0031787 | 0.0015071 |
| 47 | 0.0025365 | 0.0033855 | 0.0016416 |
| 48 | 0.0031117 | 0.0041885 | 0.0020114 |
| 49 | 0.0032814 | 0.0044154 | 0.0021277 |
| 50 | 0.0036389 | 0.0047961 | 0.0024093 |
| 51 | 0.0037549 | 0.0049154 | 0.0025192 |
| 52 | 0.0039786 | 0.0052343 | 0.0026412 |
| 53 | 0.0044125 | 0.0058018 | 0.0029677 |
| 54 | 0.0049836 | 0.0065111 | 0.0034072 |
| 55 | 0.0051789 | 0.0067560 | 0.0035424 |
| 56 | 0.0056363 | 0.0073574 | 0.0038758 |
| 57 | 0.0060944 | 0.0079217 | 0.0042305 |
| 58 | 0.0068073 | 0.0088647 | 0.0047306 |
| 59 | 0.0076663 | 0.0098937 | 0.0053974 |
| 60 | 0.0085419 | 0.0108660 | 0.0060845 |
| 61 | 0.0093770 | 0.0119552 | 0.0067040 |
| 62 | 0.0103816 | 0.0131025 | 0.0075524 |
| 63 | 0.0111210 | 0.0139892 | 0.0081584 |
| 64 | 0.0130112 | 0.0163386 | 0.0096378 |
| 65 | 0.0142114 | 0.0176561 | 0.0106747 |
| 66 | 0.0147373 | 0.0183030 | 0.0110671 |
| 67 | 0.0172294 | 0.0212806 | 0.0130651 |
| 68 | 0.0186439 | 0.0230152 | 0.0141984 |
| 69 | 0.0219140 | 0.0268936 | 0.0168179 |
| 70 | 0.0255684 | 0.0312764 | 0.0198283 |
| 71 | 0.0267284 | 0.0322916 | 0.0211461 |
| 72 | 0.0309415 | 0.0373010 | 0.0245630 |
| 73 | 0.0335910 | 0.0404687 | 0.0269002 |
| 74 | 0.0374448 | 0.0453452 | 0.0300405 |
| 75 | 0.0415111 | 0.0504941 | 0.0333061 |
| 76 | 0.0421907 | 0.0505373 | 0.0345124 |
| 77 | 0.0509685 | 0.0610141 | 0.0419861 |
| 78 | 0.0562005 | 0.0669297 | 0.0467163 |
| 79 | 0.0621214 | 0.0731964 | 0.0524711 |
| 80 | 0.0742810 | 0.0872708 | 0.0634002 |
| 81 | 0.0779065 | 0.0911711 | 0.0672525 |
| 82 | 0.0858087 | 0.0996999 | 0.0749311 |
| 83 | 0.0935184 | 0.1083552 | 0.0822866 |
| 84 | 0.1036310 | 0.1203409 | 0.0916600 |
| 85+ | 3.1970745 | 3.4148381 | 3.0841035 |

Note: Age specific mortality in China was collected from the tabulation on the 2010 population census of the People’s Republic of China, see reference [15].

**Table S8.** Age specific population in China in 2010

| Age group | Both sexes | Male | Female |
| --- | --- | --- | --- |
| 0 | 13786434 | 7461199 | 6325235 |
| 1 | 15657955 | 8574973 | 7082982 |
| 2 | 15617375 | 8507697 | 7109678 |
| 3 | 15250805 | 8272491 | 6978314 |
| 4 | 15220041 | 8246206 | 6973835 |
| 5 | 14732137 | 7988151 | 6743986 |
| 6 | 14804470 | 8034452 | 6770018 |
| 7 | 13429161 | 7292300 | 6136861 |
| 8 | 13666956 | 7423559 | 6243397 |
| 9 | 14248825 | 7726203 | 6522622 |
| 10 | 14454357 | 7830808 | 6623549 |
| 11 | 13935714 | 7522558 | 6413156 |
| 12 | 15399559 | 8288987 | 7110572 |
| 13 | 15225032 | 8161000 | 7064032 |
| 14 | 15893800 | 8463924 | 7429876 |
| 15 | 18024484 | 9524898 | 8499586 |
| 16 | 18790521 | 9795181 | 8995340 |
| 17 | 20775369 | 10760828 | 10014541 |
| 18 | 20755274 | 10744556 | 10010718 |
| 19 | 21543466 | 11079367 | 10464099 |
| 20 | 28026954 | 14201091 | 13825863 |
| 21 | 26556649 | 13357755 | 13198894 |
| 22 | 24474192 | 12281148 | 12193044 |
| 23 | 25695955 | 12876542 | 12819413 |
| 24 | 22658768 | 11292037 | 11366731 |
| 25 | 19933683 | 9969984 | 9963699 |
| 26 | 19709177 | 9879292 | 9829885 |
| 27 | 19480836 | 9801611 | 9679225 |
| 28 | 22322147 | 11271599 | 11050548 |
| 29 | 19568009 | 9914552 | 9653457 |
| 30 | 18928369 | 9604727 | 9323642 |
| 31 | 19866458 | 10141582 | 9724876 |
| 32 | 19474874 | 9909833 | 9565041 |
| 33 | 18179478 | 9289224 | 8890254 |
| 34 | 20689024 | 10576456 | 10112568 |
| 35 | 21186516 | 10817432 | 10369084 |
| 36 | 22906980 | 11690644 | 11216336 |
| 37 | 23990208 | 12283353 | 11706855 |
| 38 | 24730460 | 12662559 | 12067901 |
| 39 | 25211795 | 12937116 | 12274679 |
| 40 | 27397219 | 13993123 | 13404096 |
| 41 | 24956297 | 12723691 | 12232606 |
| 42 | 27032542 | 13782610 | 13249932 |
| 43 | 21355748 | 10856214 | 10499534 |
| 44 | 24012158 | 12253040 | 11759118 |
| 45 | 23962574 | 12252515 | 11710059 |
| 46 | 23355778 | 11867147 | 11488631 |
| 47 | 26972157 | 13803796 | 13168361 |
| 48 | 20075084 | 10224798 | 9850286 |
| 49 | 11228960 | 5628162 | 5600798 |
| 50 | 14097008 | 7205176 | 6891832 |
| 51 | 12838832 | 6624865 | 6213967 |
| 52 | 16617709 | 8570000 | 8047709 |
| 53 | 18351980 | 9422827 | 8929153 |
| 54 | 16847642 | 8540366 | 8307276 |
| 55 | 17610528 | 8973192 | 8637336 |
| 56 | 17738127 | 8981235 | 8756892 |
| 57 | 16093888 | 8099033 | 7994855 |
| 58 | 16167933 | 8153588 | 8014345 |
| 59 | 13701998 | 6875890 | 6826108 |
| 60 | 13618204 | 6917026 | 6701178 |
| 61 | 13029125 | 6690003 | 6339122 |
| 62 | 11276853 | 5719180 | 5557673 |
| 63 | 10791633 | 5492805 | 5298828 |
| 64 | 9951467 | 5015412 | 4936055 |
| 65 | 9073411 | 4564266 | 4509145 |
| 66 | 8640965 | 4391409 | 4249556 |
| 67 | 7942141 | 4003493 | 3938648 |
| 68 | 7740868 | 3904424 | 3836444 |
| 69 | 7715897 | 3884879 | 3831018 |
| 70 | 7389412 | 3724605 | 3664807 |
| 71 | 6265718 | 3116177 | 3149541 |
| 72 | 6893225 | 3449237 | 3443988 |
| 73 | 6343869 | 3149307 | 3194562 |
| 74 | 6080173 | 2964127 | 3116046 |
| 75 | 5632477 | 2690547 | 2941930 |
| 76 | 5175500 | 2454168 | 2721332 |
| 77 | 5082383 | 2420196 | 2662187 |
| 78 | 4254858 | 1983724 | 2271134 |
| 79 | 3706915 | 1730224 | 1976691 |
| 80 | 3737259 | 1716514 | 2020745 |
| 81 | 2816693 | 1257795 | 1558898 |
| 82 | 2757918 | 1212683 | 1545235 |
| 83 | 2237138 | 964710 | 1272428 |
| 84 | 1824190 | 765800 | 1058390 |
| 85+ | 7580214 | 2848398 | 4731816 |

Note: Age specific population in China was collected from the tabulation on the 2010 population census of the People’s Republic of China[15].

**Table S9.** Age specific incidence rate of esophagus cancer in China in 2012(1/100 000)

| Age group | Both sexes | Male | Female |
| --- | --- | --- | --- |
| 0 | 0.0000000 | 0.0000000 | 0.0000000 |
| 1 | 0.0000000 | 0.0000000 | 0.0000000 |
| 5 | 0.0000000 | 0.0000000 | 0.0000000 |
| 10 | 0.0000000 | 0.0000000 | 0.0000000 |
| 15 | 0.0000001 | 0.0000002 | 0.0000000 |
| 20 | 0.0000006 | 0.0000002 | 0.0000011 |
| 25 | 0.0000015 | 0.0000015 | 0.0000014 |
| 30 | 0.0000032 | 0.0000037 | 0.0000028 |
| 35 | 0.0000098 | 0.0000141 | 0.0000053 |
| 40 | 0.0000295 | 0.0000441 | 0.0000143 |
| 45 | 0.0001004 | 0.0001624 | 0.0000358 |
| 50 | 0.0001884 | 0.0002976 | 0.0000733 |
| 55 | 0.0004465 | 0.0006790 | 0.0002072 |
| 60 | 0.0007459 | 0.0010847 | 0.0004037 |
| 65 | 0.0010406 | 0.0014843 | 0.0005984 |
| 70 | 0.0012591 | 0.0017736 | 0.0007643 |
| 75 | 0.0013875 | 0.0019303 | 0.0009053 |
| 80 | 0.0014068 | 0.0019315 | 0.0009907 |
| 85+ | 0.0012141 | 0.0017703 | 0.0008822 |

Note: Age specific incidence rate of esophagus cancer in China in 2012 collected from published paper of the Mortality and survival analysis of esophageal cancer in China[6].

**Table S10.** Age specific mortality of esophagus cancer in China in 2012(1/100 000)

| Age group | Both sexes | Male | Female |
| --- | --- | --- | --- |
| 0 | 0.0000000 | 0.0000000 | 0.0000000 |
| 1 | 0.0000000 | 0.0000000 | 0.0000000 |
| 5 | 0.0000000 | 0.0000000 | 0.0000000 |
| 10 | 0.0000000 | 0.0000000 | 0.0000000 |
| 15 | 0.0000001 | 0.0000002 | 0.0000000 |
| 20 | 0.0000006 | 0.0000002 | 0.0000011 |
| 25 | 0.0000015 | 0.0000015 | 0.0000014 |
| 30 | 0.0000032 | 0.0000037 | 0.0000028 |
| 35 | 0.0000098 | 0.0000141 | 0.0000053 |
| 40 | 0.0000295 | 0.0000441 | 0.0000143 |
| 45 | 0.0001004 | 0.0001624 | 0.0000358 |
| 50 | 0.0001884 | 0.0002976 | 0.0000733 |
| 55 | 0.0004465 | 0.0006790 | 0.0002072 |
| 60 | 0.0007459 | 0.0010847 | 0.0004037 |
| 65 | 0.0010406 | 0.0014843 | 0.0005984 |
| 70 | 0.0012591 | 0.0017736 | 0.0007643 |
| 75 | 0.0013875 | 0.0019303 | 0.0009053 |
| 80 | 0.0014068 | 0.0019315 | 0.0009907 |
| 85+ | 0.0012141 | 0.0017703 | 0.0008822 |

Note: Age specific mortality of esophagus cancer in China in 2012 collected from published paper of the Incidence and trend analysis of esophageal cancer in China[5].

**Table S11.** The predicted incidence per 100 000 of esophageal cancer from model simulation

and model inputs

| Year | Model prediction | Model inputs* |
| --- | --- | --- |
| 2013 | 20.84 | 20.98 |
| 2014 | 20.69 | 20.79 |
| 2015 | 20.54 | 20.60 |
| 2016 | 20.40 | 20.42 |
| 2017 | 20.26 | 20.23 |
| 2018 | 20.12 | 20.05 |
| 2019 | 19.98 | 19.87 |
| 2020 | 19.85 | 19.69 |
| 2021 | 19.71 | 19.52 |
| 2022 | 19.58 | 19.34 |
| 2023 | 19.46 | 19.17 |
| 2024 | 19.34 | 18.99 |
| 2025 | 19.22 | 18.82 |
| 2026 | 19.08 | 18.65 |
| 2027 | 18.97 | 18.49 |
| 2028 | 18.85 | 18.32 |
| 2029 | 18.74 | 18.15 |
| 2030 | 18.63 | 17.99 |

Note: * predicted based on the population-based cancer registry of China from 2000 to 2011
